# Dentist’s Manual of Special Chemistry

**Published:** 1888-04

**Authors:** 


### The Dental College Series of Text Books.

### The Dentist's Manual of Special Chemistry.

BY CLIFFORD MITCHELL, A. B. [HARV.] M.D.

This work, just from press, published by the author, is designed as a text book for the dental student and a book of reference for the dental practitioner as well, and from a hasty glance at its contents, and the manner of arrangement, we have no hesitancy in saying that it is admirably adapted to the purposes for which it was prepared. It is different from most other works of this character, in that it presents in a most condensed form that which is essential to the student. Its statements are so well and so clearly made, as to be especially attractive to the searcher after chemical knowledge. Too often in text books there is a large amount of verbiage that rather detracts from the value of the work as a text book. Evidently the intention of the author of this work, was not to make a large book, but to see how much of that which is essential could be crowded into the smallest possible space. This book should be adopted as a text book by all college faculties. It should be in the hands of every student, and in the library of every dentist, and especially of those who lay any claims to the science of our profession. The work was prepared under the supervision of Dr. A. O. Hunt, D.D.S., Professor of Chemistry and Metallurgy, in the Dental Department of the University of Iowa, which is an additional gaurantee as to the character and value of the work. It is published at $2.25 per copy, 20 per cent. discount to dental students and practitioners, and can douhtless be obtained of book-sellers generally.

